# Impact of surface topography on biofilm formation by *Candida albicans*

**DOI:** 10.1371/journal.pone.0197925

**Published:** 2018-06-18

**Authors:** Katherine Lagree, Htwe H. Mon, Aaron P. Mitchell, William A. Ducker

**Affiliations:** 1 Department of Biological Sciences, Carnegie Mellon University, Pittsburgh, PA, United States of America; 2 Department of Chemical Engineering, and Center for Soft Matter and Biological Physics, Virginia Tech, Blacksburg, VA, United States of America; Institute of Microbiology, SWITZERLAND

## Abstract

*Candida albicans* is a fungal pathogen that causes serious biofilm-based infections. Here we have asked whether surface topography may affect *C*. *albicans* biofilm formation. We tested biofilm growth of the prototypical wild-type strain SC5314 on a series of polydimethylsiloxane (PDMS) solids. The surfaces were prepared with monolayer coatings of monodisperse spherical silica particles that were fused together into a film using silica menisci. The surface topography was varied by varying the diameter of the silica particles that were used to form the film. Biofilm formation was observed to be a strong function of particle size. In the particle size range 4.0–8.0 μm, there was much more biofilm than in the size range 0.5–2.0 μm. The behavior of a clinical isolate from a clade separate from SC5314, strain p76067, showed results similar to that of SC5314. Our results suggest that topographic coatings may be a promising approach to reduce *C*. *albicans* biofilm infections.

## Introduction

*Candida albicans* is a widespread opportunistic fungal pathogen [1]. It colonizes mucosal surfaces of the human body such as the oral cavity and gastrointestinal tract, where it is generally a benign commensal. However, colonization makes *C*. *albicans* available to form biofilms on implanted medical devices such as urinary catheters or intravenous catheters. These biofilms serve as a source of *C*. *albicans* cells that disseminate through the bloodstream to cause invasive candidiasis [2]. The biofilm growth form of *C*. *albicans*, like that of most bacteria, is recalcitrant to treatment with many conventional antimicrobials [2]. Therefore, interventions that inhibit biofilm formation on medical devices hold promise to reduce the frequency of device-associated biofilm infections.

One strategy for preventing device-associated biofilm infections is to use surface coatings to limit the organism’s ability to adhere to solid surfaces. Coatings technology is useful because once a suitable coating is found to reduce biofilm formation, then that coating can be applied to a variety of products, obviating the need to develop a new anti-biofilm solution for each product. Adherence to solids can be reduced in several ways that can be broadly classified as either chemical or physical. Chemical coatings, e.g. polyethylene oxide brushes, are known to reduce attachment of small molecules and also a variety of bacteria [3–8]. Chemical coatings can also contain antimicrobials that kill bacteria [6, 9]. One limitation of chemical coatings is that their effects can be mitigated by adsorption of very thin (molecular) contaminating films [10]. A limitation of antimicrobials is that they often have to leach out from a solid [6], having undesirable impact remote from the source, and also eventually becoming depleted. Our focus here is the action of a physical coating–a coating of microscopic spheres–to reduce adherence by presenting an unattractive topography to the microorganism. These coatings do not incur the limitations described above. Topographic coatings have previously been investigated for their action against bacteria [9, 11–24] and against marine organisms [25]. There has also been some investigation of the effects of topography on *C*. *albicans*. Verran and Maryan [26] found that *C*. *albicans* is more likely to attach to scratch marks and pits on surfaces that were scratched with emery paper. Whitehead et al [27] found that, in contrast to a selection of bacteria, the number of *C*. *albicans* retained after rinsing was *not* significantly changed by arrangements of surface pits in the size range 0.2–2 μm. Very recent work by Alalwan *et al*. [28] showed that a square pattern of 120 nm diameter pits did reduce the number of *C*. *albicans* on a solid compared to a flat solid. A hexagonal or non-square arrangement did not have an effect. Research has also shown the TiO_2_ coatings can increase the growth of C. albicans [29], but TiO_2_ nanoparticles can decrease the growth [30].

One aspect of prior work is that it draws attention to complexity of surface topography as a parameter. Some work uses surface roughness parameters, whereas diverse features can have the same surface roughness. The work by Alalwan et al is an example of how the arrangement of features can be important and the work of Verrran and Maryan demonstrates that inhomogeneities, e.g. scratches and pits can be very important. Here we focus on very homogeneous coatings with uniform topography.

**Fig 1:**
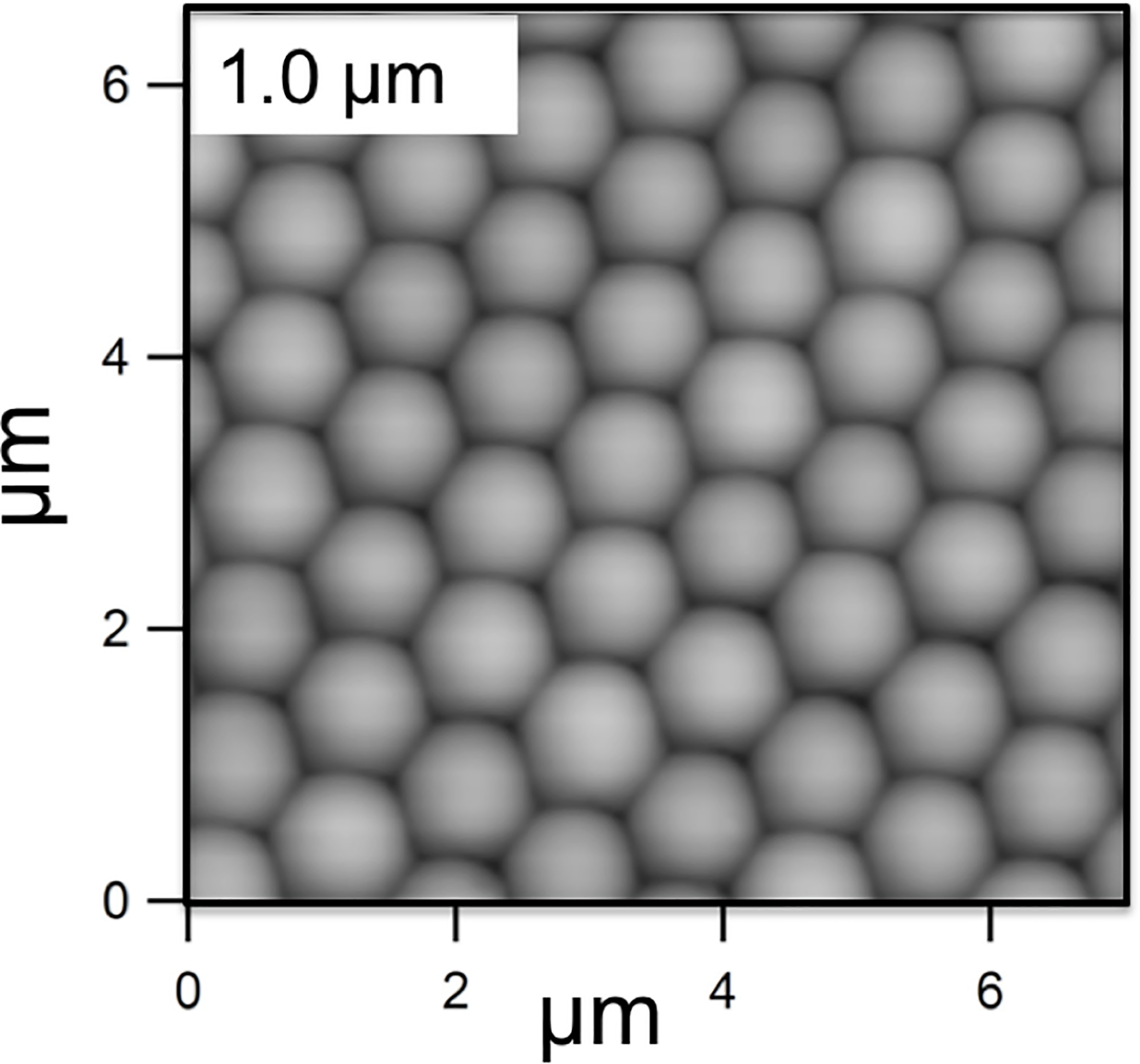
Atomic Force Microscope image of test surface consisting of 1 μm diameter silica spheres adhered to a PDMS solid. The entire sample is coated in a very thin layer of silica.

In this paper we investigate the effects of topography on biofilm formation by the fungal pathogen *C*. *albicans*. Coatings of colloidal particles (see Fig 1) are used to vary the topography by varying the diameter of the particles. Crystalline monolayers of colloidal particles are known as colloidal crystal monolayers and a method of producing robust films from monolayers of silica (SiO_2_) colloidal crystals has been published previously [31]. Colloidal crystal monolayers with particle dimensions 0.5–8 μm have been shown to reduce the adsorption and biofilm development of the bacterium *Pseudomonas aeruginosa* [12, 24, 32, 33]. The particle diameter was similar to the dimensions of the bacteria, a rod of diameter 1 μm and length 2–3 μm. *C*. *albicans* cells are much larger: yeast cells have approximately 5 μm diameter, and hyphae chains of 2 μm diameter rod-shaped cells can reach over 500 μm in length [34]. Therefore we have investigated a larger range of particles sizes (0.5–8.0 μm) so that the particle size approaches the organism size. The coatings we investigated here consist of a close-packed layer of silica particles that are attached to the polymer, polydimethylsiloxane (PDMS), which is a form of silicone rubber. Silicone is commonly used in medical devices [35], for example, many catheters are molded from silicone [36–40]. This film has been shown to be (a) easy to manufacture, and (b) robust [31], both of which should enhance the prospect of applications. We examine *C*. *albicans* strain SC5314, because it is the canonical lab strain from which almost all current genetic, molecular, and phenotypic knowledge of *C*. *albicans* has been obtained, and a second biofilm-forming strain, p57055, has been chosen because it is from a different clade (Clade II) than SC5314 (Clade I). We show that there is a particle-size dependent effect on the adherence of *C*. *albicans* for both strains and that the optimal particle size to deter adherence on *C*. *albicans* is similar to that for *P*. *aeruginosa*. We find that overexpression of a major biofilm adhesin gene, *ALS1*, does not overcome the impediment imposed by surface topography. Our results support the idea that surface topography manipulation may provide an effective biofilm deterrent for implanted devices.

## Experimental

### Fabrication of test solids

**Fig 2:**
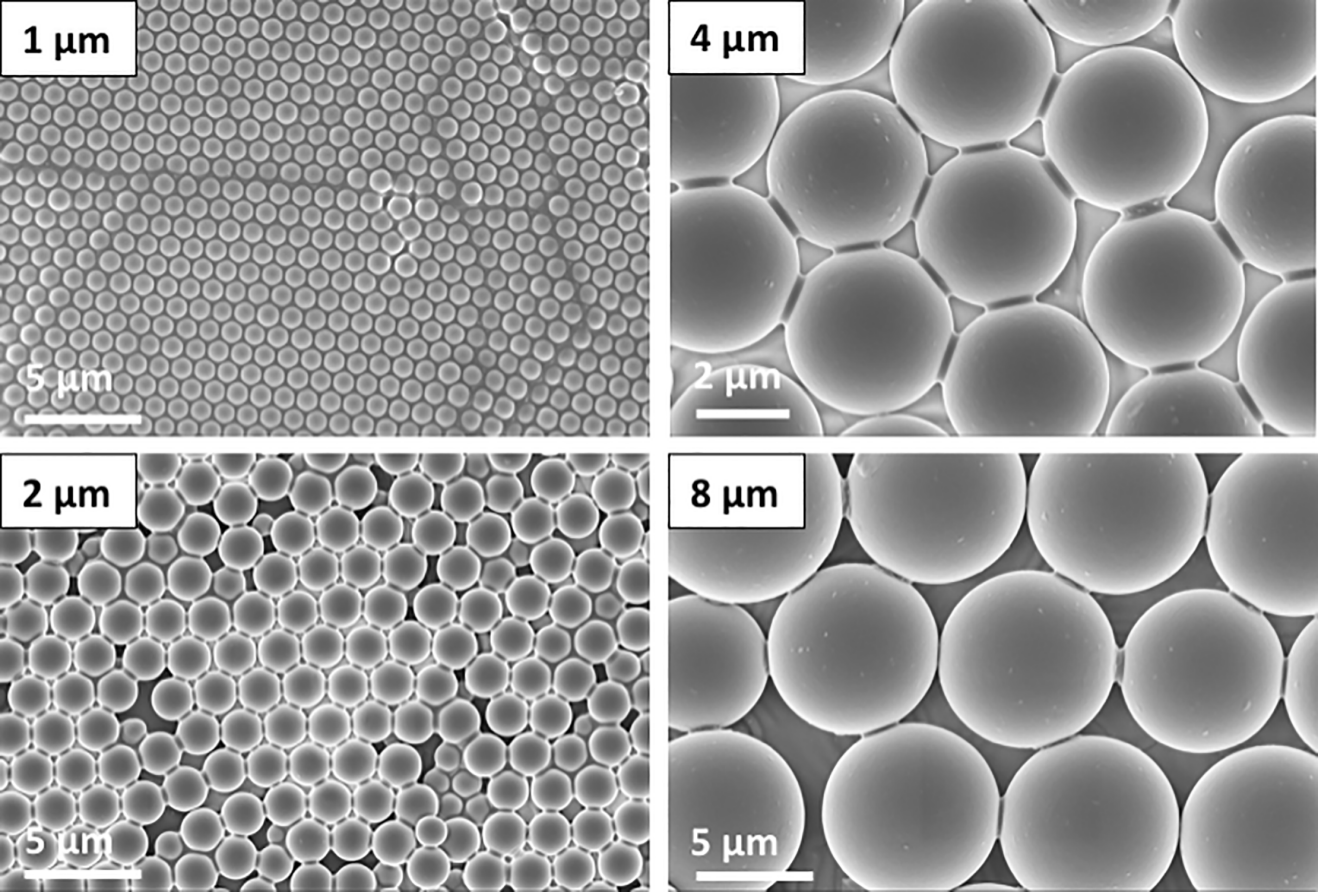
Scanning electron microscope (SEM) images of films fabricated from the 1, 2, 4, and 8 μm diameter silica spheres. The small bridges between the particles are evident in the images of the 4 and 8 μm particles. These bridges, together with similar bridges to the solid, stabilize the film so that it is unaffected by exposure to a stirred solution, rinsing etc.

The test solids, which were the “substratum” for the biofilm, consisted of PDMS to which a monolayer of spherical colloidal particles was added. The monolayer of particles was bound to each other and to the PDMS by silica produced by a sol-gel technique. Fabrication is described in brief here and in more detail elsewhere [31]. The PDMS was prepared from Sylgard 184 components (Dow Corning, MI) and cured in a petri dish. The PDMS was heated at 150°C for 48 h to evaporate unreacted components. Monodisperse dry powder of silica spheres (0.5, 1.0, 2.0, 4.0 or 8.0 μm purchased from the Fiber Optic Center Inc., MA) was deposited onto the PDMS, and arranged into a crystalline lattice by rubbing with a second piece of PDMS. The formation of the crystal was confirmed by laser light scattering. Each sample was treated with O_2_ plasma at 100 W for 1 min (SPI Supplies, PA) to form reactive hydroxyl groups to enhance bonding, and then the arrangement of particles was fixed using a solution of tetraethoxysilica (TEOS), NH_4_OH, H_2_O, and ethanol to form menisci between the particles and between the particles and the PDMS. The resulting film was stable when immersed in salt solutions. The final structure of each test solid was confirmed by AFM (0.5 μm and 1.0 μm particles) or by light microscopy (2.0, 4.0, and 8.0 μm particles). A sample with no particles was also included. This consisted of a PDMS without a monolayer of colloidal particles, which was exposed to O_2_ plasma and TEOS in the same way as the samples with particles and is labelled as “0 μm” in the figures. Fig 1 shows an Atomic Force Microscope (AFM) image of the surface of a 1 μm—particle film recorded with a Asylum Research Cypher model, and films of the larger-diameter larger particles and are shown in Fig 2.

### Biofilm growth

Strains were grown overnight in YPD (1% Difco yeast extract, 2% Bacto-peptone, 2% dextrose) at 30°C in a rotating drum. Biofilms were grown as previously described with slight modifications [41]. Surfaces were placed in a 12 well dish and 2mL of RPMI (Roswell Park Memorial Institute 1640 medium) were added at 37°C. Strains were inoculated at an optical density at 600 nm (OD) of 0.2 and incubated in a 37°C orbital shaker at 60 RPM. The cells were allowed to adhere for 90 minutes. The media was removed and 2 mL of phosphate buffered saline (PBS) were carefully added to remove the unadhered cells. The PBS was aspirated and 2 mL of fresh RPMI were added. The biofilms were grown for 48 hours, fixed, and stained for imaging.

### Confocal microscopy

Biofilms were fixed and imaged as previously described [41]. Briefly, biofilms were fixed with 4% formaldehyde and 2.5% glutaraldehyde for 1 hour. Fixed biofilms were washed with PBS and stained with ConA Alexa Fluor 594 Conjugate in phosphate buffered saline (PBS) for 24 hours. Fixed and stained biofilms were dehydrated and optically cleared using 100% methanol, followed by 50:50 methanol: methyl salicylate then 100% methyl salicylate. Cleared biofilms were inverted onto a silicone ring (300μm) on an engineered stage insert that consisted of a cover glass cemented to a black-anodized aluminum stage insert. Approximately 200 μL methyl salicylate was added to the engineered stage insert to float the biofilm, while still maintaining contact with the silicone ring through surface tension. Biofilms were imaged using a slit-scanning confocal optical unit on a Zeiss Axiovert 200 microscope. A 40x 0.85-numerical aperture oil immersion objective was used to image the biofilms. Optical sections were collected with a step size of 0.9 μm in a series of 130 planes for a total of 225 planes. Stacks were concatenated and background subtracted using FIJI [42]. Side views were created by reslicing the stack and using the z-project function with maximum intensity. The Yellow Hot lookup table was applied to the final side view image. Apical views were created by using the Temporal-Color Code plugin with the Spectrum lookup table.

### Quantification of biofilm volume

The biovolume was determined by analysis of scanning confocal microscopy images. After growth for 48 h in RPMI medium, the test solids were removed from growth medium, gently washed, fixed, stained, dehydrated, and optically cleared. The biovolume was measured using COMSTAT.[43] This analysis measures the total volume of pixels per area parallel to the test solid surface that have an intensity greater than a specified threshold. This volume per area is thus representative of an average thickness. For each particle size, 3 different positions were examined on one sample.

## Results

**Fig 3:**
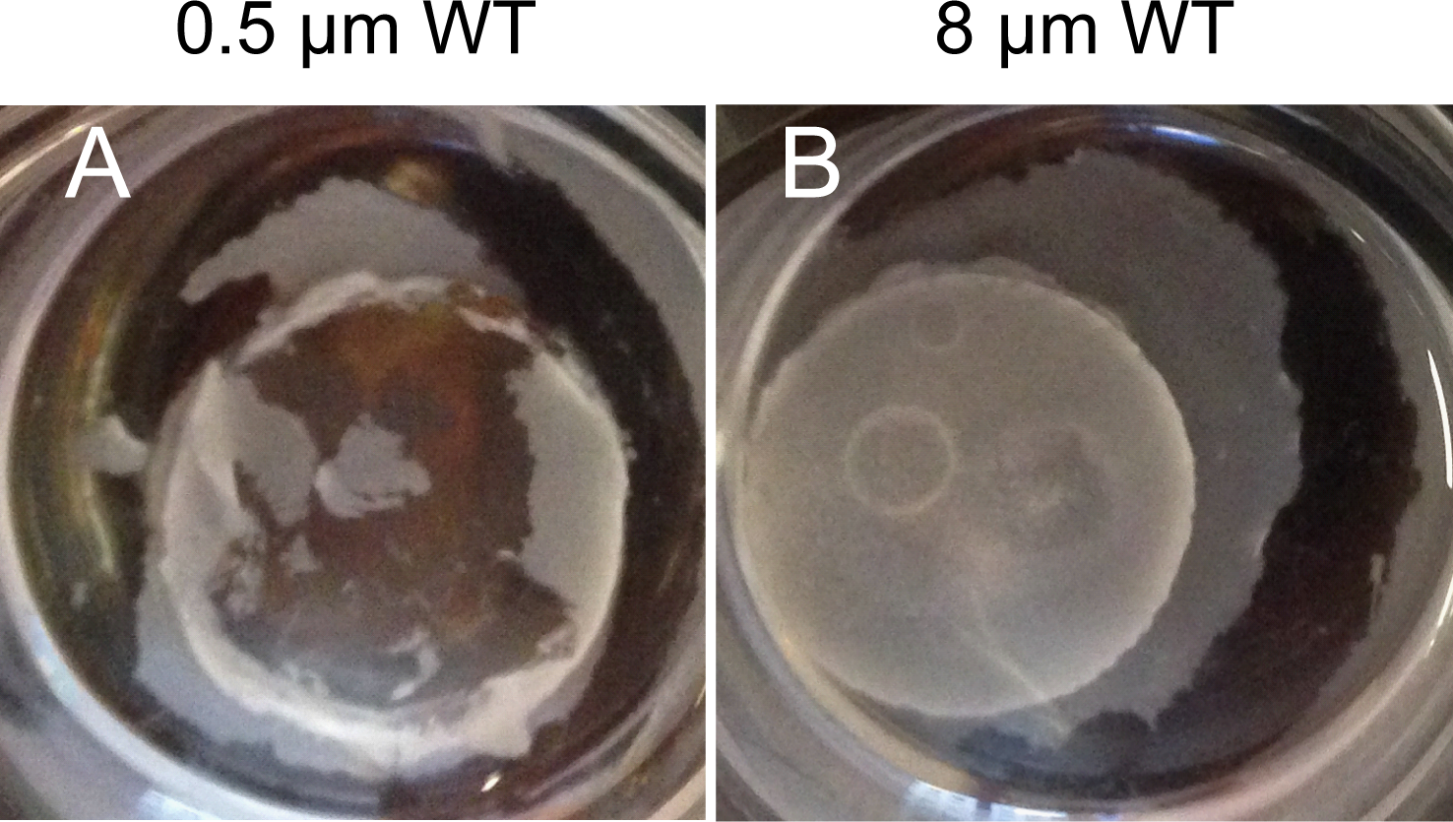
Photographs of *C*. *albicans* grown in (RPMI) media for 24 hours, 37°C on test solids as indicated.

**Fig 4:**
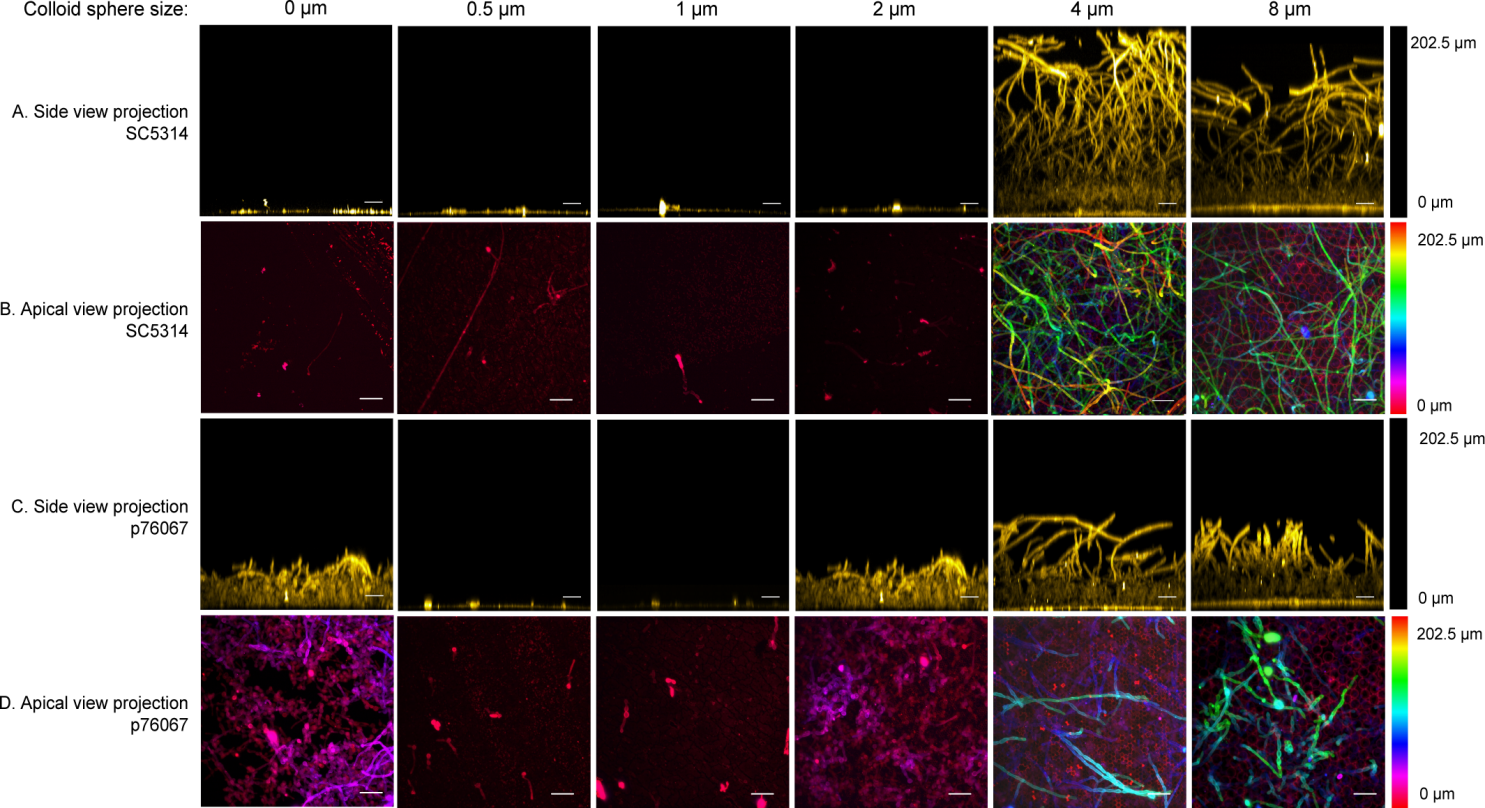
Confocal images of biofilm growth on colloid crystal surfaces. Wild-type *C*. *albicans* strains SC5314 (rows A, B) and p76067 (rows C, D) were grown on test solids with colloid crystal sphere diameters indicated above each column, then fixed and stained with ConA Alexa Fluor 594 as detailed in Methods. Rows A and C show side view projections; rows B and D show apical projections. The scale bar corresponds to 20μm. The hyphae are the long fibrous features; the yeast cells the ~3μm oval features. The 0 μm sample refers to PDMS with a layer of silica grown on it. This solid has nanometer-scale roughness but no micrometer-scale features. In this sense, it is equivalent to a coating of zero-μm spheres.

To determine whether surface topography can alter biofilm formation by *C*. *albicans*, we used a panel of colloidal crystal monolayers with particle diameters of 0.5, 1.0, 2.0, 4.0, or 8.0 μm. Biofilm growth was examined at 48 hr after inoculation with wild type strain SC5314. There was considerable growth in the liquid medium for all samples (illustrated in Fig 3A and 3B), which indicates that none of the solids leached compounds that were sufficiently toxic to inhibit *C*. *albicans* growth. We observed that SC5314 failed to form a biofilm on the coated PDMS formulation alone (Fig 4, rows A, B), thus indicating that biofilm adherence would be confined to the colloid crystal surfaces. Biofilm formation was evident on colloidal crystal monolayers with particle diameters of 4.0 or 8.0 μm. Biofilm depth on these surfaces was 150–200 μm (Fig 4, row A), and hyphae were abundant in each biofilm (Fig 4 rows A, B). Strikingly, though, biofilm formation was severely reduced on colloidal crystal monolayers with particle diameters of 0.5, 1.0, or 2.0 μm (Fig 4 row A). Few cells remained attached to these surfaces (Fig 4 row B). The presence of large cell aggregates suspended in the growth medium of all samples suggests that there is functional cell–cell adherence, but that cell–solid adherence is diminished for the 0.5, 1.0, or 2.0 μm solids (Fig 3A). These observations indicate that surface topography is a critical determinant of stable *C*. *albicans* biofilm formation capability on solids.

**Fig 5:**
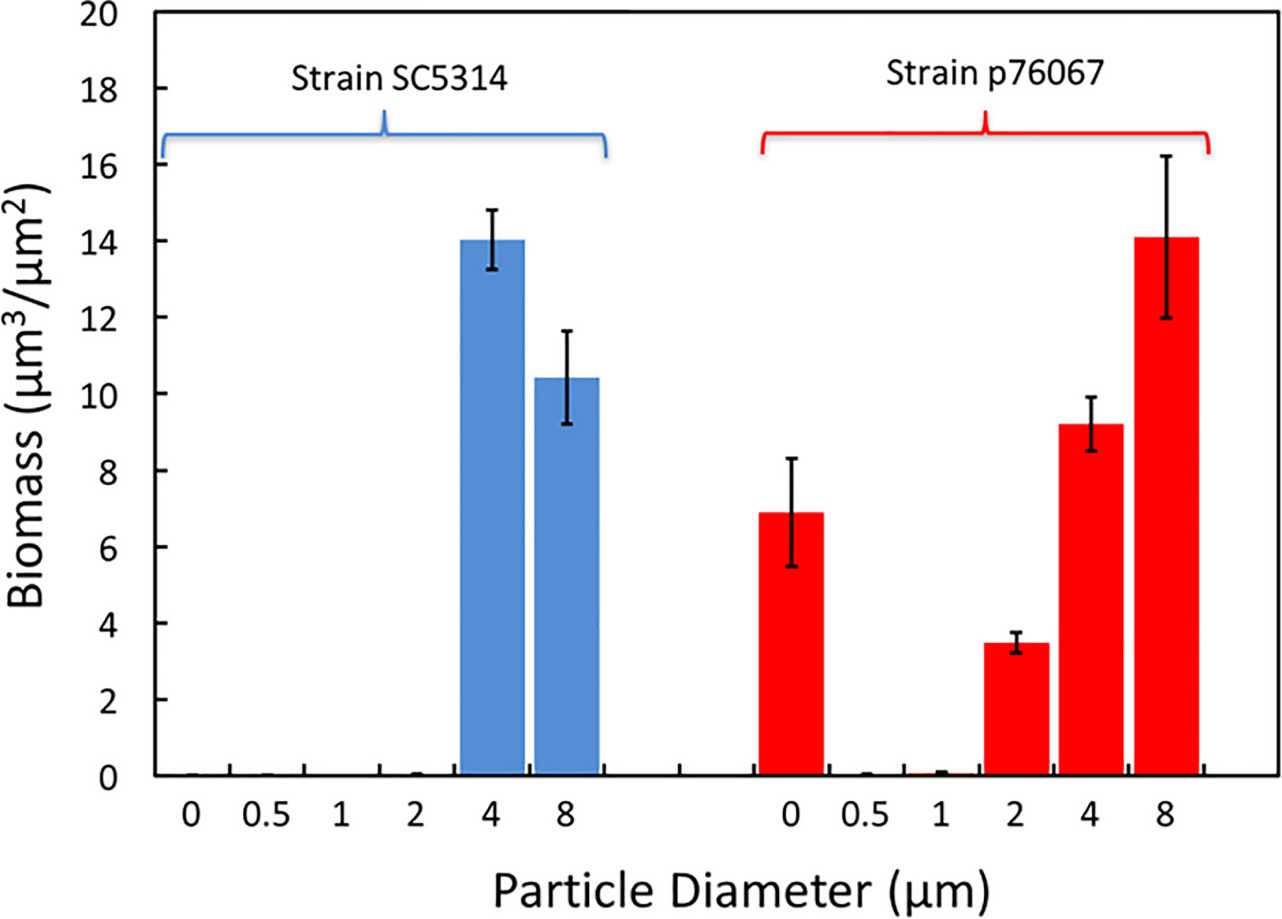
Biofilm volume, as determined from COMSTAT analysis of confocal images, as a function of the diameter of the colloidal particles for two strains of *C*. *albicans*. Zero represents a PDMS sample with a thin coating of silica. The 4 or 8 μm samples have significantly more biofilm than the 0.5 or 1 μm samples. Data are means from three positions on one sample of each strain, and the error bars are plus and minus the standard error. 0-μm represents the silica-coated PDMS.

The biovolume of cells on the various solids was quantified from confocal imaging data using COMSTAT, and the results are shown in Fig 5. The data confirm that there is very little biofilm on the solids coated with no particles, 0.5, 1.0, and 2.0 μm particles compared to those coated with 4.0 and 8.0 μm particles. There is a distinct cut-off in behavior for diameters between 2 and 4 μm: the biofilm volume on the small particles is at least 500 times lower than on the larger particles.

**Fig 6:**
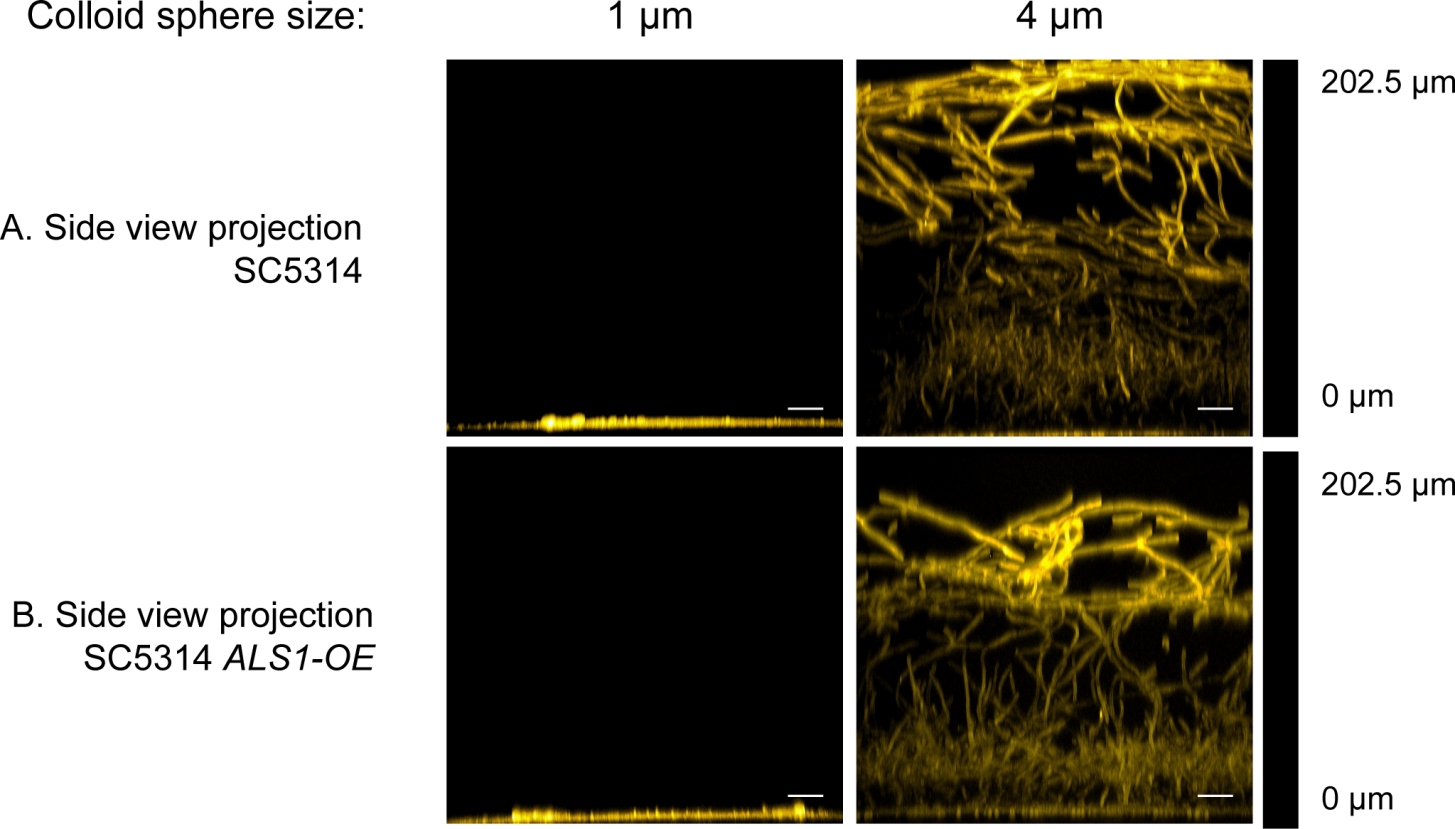
Confocal images of biofilm growth of strains SC5314 and *ALS1-OE* on colloid crystal surfaces. *C*. *albicans* strains SC5314 (row A) and *ALS1-OE* (row B) were grown on test solids with colloid crystal sphere diameters of 1 μm and 4 μm, as indicated above each column, then fixed and stained with ConA Alexa Fluor 594 as detailed in Methods. Images are side-view projections. The scale bar corresponds to 20μm.

We hypothesized that prohibitive surface topography may impose a defect in surface adherence. Therefore, it seemed possible that overexpression of a biofilm adhesin in *C*. *albicans* cells might overcome prohibitive surface topography. Prior studies indicate that *ALS1* is a major adhesin gene [2] that functions in both yeast cells as well as hyphae [44, 45] We used a derivative of SC5314, called *ALS1-OE*, that expresses *ALS1* from the *TDH3* promoter [17]. Strain *ALS1-OE*, like SC5314, formed biofilms on 4.0 μm-diameter colloidal crystal monolayers (Fig 6A and 6B). However, *ALS1-OE* was unable to form a biofilm on 1 μm-diameter colloidal crystal monolayers, and thus shared the biofilm defect observed with SC5314 on this surface (Fig 6A and 6B). Our results indicate that increased expression of a biofilm adhesin gene does not overcome prohibitive surface topography.

SC314 is often the only strain of *C*. *albicans* that is investigated, but we wanted to test a second clinical isolate, strain p76067, to determine whether surface topography effects were strain-specific. Strain p76067 belongs to *C*. *albicans* clade II, which represents a divergent genotype from the clade I strains represented by SC5314, and was chosen because it is capable of robust biofilm formation [46]. Strain p76067 was able to form a biofilm on silica-coated PDMS alone (Fig 4, row C, D), unlike SC5314. Strain p76067 formed biofilms on 4.0 or 8.0 μm-diameter colloidal crystal monolayers as well. Biofilm depth was roughly 100 μm (Fig 4, row C), and biomass was comparable to that of SC5314 biofilms on the respective surfaces (Fig 5). However, as observed with SC5314, biofilm formation and biomass were severely reduced on 0.5 or 1.0 μm-diameter colloidal crystal monolayers (Fig 4, row C; Fig 5) compared to the 2, 4, or 8 μm samples. Interestingly, p76067 biofilm formation was partially impaired on the 2.0 μm-diameter colloidal crystal monolayer (Fig 4, row C; Fig 5). This quantitative defect was associated with a qualitative change in appearance: while biofilms on 4.0 or 8.0 μm-diameter surfaces showed presence of hyphae, the biofilms on 2.0 μm-diameter surfaces comprised largely yeast-form cells.

Combining the data for both strains, Tukey’s multiple comparison test shows that the results fall into two distinct groups: results for each of 0.5 and 1.0 μm are significantly different (*p*<0.05) than those of either 4 or 8 μm particles, and there are no significant differences within the two groups. Comparison between 2 μm and either 4 or 8 μm fall just outside the normal level for significance (0.051 and 0.058, respectively). Overall, our observations indicate that 0.5 or 1.0 μm-diameter colloidal crystal monolayers are prohibitive for biofilm formation by either of the two *C*. *albicans* strains.

## Discussion

Our findings here support the idea that surface topography can influence *C*. *albicans* biofilm attachment to a solid. The most important observation is that some topographies are prohibitive for biofilm attachment, because this result suggests the possibility that a designed topography on implanted medical devices could reduce *C*. *albicans* biofilm formation on implants in patients. Although prior studies have indicated that there are prohibitive topologies for bacteria, we note that *C*. *albicans* filaments can have lengths that are many times that of bacteria. Therefore, it seemed possible that impaired adherence observed with bacteria might not translate to *C*. *albicans* because adhesion forces depend on the size of objects, or because there is a required match in dimension between the topography and the microbe. Our results show that surface biofilm formation by *C*. *albicans* in vitro can be prohibited despite the presence of filaments and the different size scale of the organism (5 μm vs 3 μm).

Why do some surface topographies fail to support biofilm formation? Our hypothesis to explain the results for *C*. *albicans* is based on the observation that there are always biofilm-like mat fragments that are nonadherent present in the culture containing the surfaces that prohibit biofilm formation. We propose that prohibitive topologies reduce cell–surface adhesion strength, just as observed for bacteria. However, filamentous cells of *C*. *albicans* have a high degree of cell–cell adhesion as well. Thus, prohibitive surfaces reduce the ratio of cell–surface to cell–cell adhesion strength. An analogous circumstance was studied by d'Enfert and colleagues[18] with a strain that overexpressed the adhesin gene *PGA22*. This strain was unable to form a mature biofilm because the adhesin caused augmented cell clustering, and the clusters in turn displayed increased sensitivity to shear forces. We propose that the situation with prohibitive topologies is similar, in that cell–cell adhesion strength remains strong enough that a surface-bound biofilm would be subject to a large shear stress from fluid flowing past cell clusters while at the same time having a weakened surface attachment due to the topography. Therefore, overexpression of the *ALS1* adhesin gene could not restore biofilm formation because it increases both cell-cell and cell-substrate adherence, thus maintaining susceptibility to removal by shearing. Importantly, this model explains the surprising biofilm phenotype of strain p76067 on 2.0 μm-diameter surfaces: yeast cells with weak cell–cell adherence are retained on the surface, whereas filamentous cells with strong cell–cell adherence are not. It seems likely that the filamentous cells are sheared off, and continued growth of yeast cells fills in the gaps.

At this stage it is unclear what the mechanism is by which coatings of spheres or other topographies inhibit microbial adhesion. Several hypotheses have been advanced:

1. The topography may present fewer binding sites than a flat surface [25].
2. Adhesion may be more difficult on regions where the solid has a similar curvature to the microorganism [9, 12, 47]. This was originally based on the ideas from the literature on liposomes: that adhesion to a curved surface incurs a bending energy penalty [48].
3. Topography may trap air at the solid (the Cassie state), which in turn reduces access of the microorganism to the solid [15]. However, topographic reductions in bacterial growth have also been reported on hydrophilic solids [12].

Our results for *C*. *albicans* can not be explained by the existence of the Cassie state. Although the surface film has a high contact area, it has a contact angle approaching zero (it is completely wet by water). The results also do not neatly fit into the size match hypothesis. The yeast cells are approximately 5 μm in diameter but the yeast cells are more inhibited by the topography of size 0.5–1 μm than by the 4–8 μm topography. For strain p76067, the 2 μm particles did inhibit the 2 μm diameter hyphae but not the 5 μm cells, but such size matching was not universal to our results. What remains is the hypothesis that the topography provides fewer binding sites. However, this is a rather incomplete hypothesis. It is not clear why the number should matter more than the quality of binding site.

It is very convenient from a technological point-of-view that the particle diameters that inhibit *C*. *albicans* growth also inhibit *P*. *aeruginosa* growth: particles of 1 μm work well against both organisms. Importantly, we validated their effectiveness with two biofilm-forming *C*. *albicans* strains. In future studies it will be important to test these surfaces with in vivo biofilm infection models, in which biofilms are reinforced by host proteins and shear forces may fluctuate. Similarly, it will be important to test these surfaces with other fungal species such as *Candida glabrata* and *Candida parapsilosis* which can form robust mixed biofilms with *Candida albicans*. Nonetheless, at this stage our results suggest that there are broad spectrum anti-biofilm topographies that could reduce device-associated infection in patients.

Finally, we note that the topographical approach to hindering biofilms that we have described here has a number of useful qualities. First, it is a coating. Once suitable antifouling properties have been demonstrated, the same coating can be applied to multiple objects. Second, the coating procedure is relatively simple and inexpensive: the coating is simply a monolayer of spheres that are already mass produced, and the coating procedure used here was simply to rub the particles onto the solid in a process that has some similarity to painting with latex paints. The coating does not require expensive beam nanofabrication or flat surfaces. Finally, the fabrication technique can be used for a variety of particle sizes to tailor the scale of the topography to particular applications.

## Conclusions

The adherence of *C*. *albicans* biofilm (SC5314 or p76067) is very sensitive to surface topography for the conditions studied here. When we coat a solid with spherical particles, the biofilm volume remaining after gentle rinsing is at least 100 times lower on 0.5 or 1.0 μm particles than on 4 and 8 μm particles and is near or below our level of detection. Overexpression of the Als1 adhesin is not sufficient to improve adhesion of *C*. *albicans* SC5314 on 0.5 μm particles. The sensitivity of adherence to topography suggests that topographic coatings may be useful in combating *C*. *albicans* infections. Possible applications include coatings applied to medical implants such as catheters or household items that are routes of transfer between humans.
